# Antimicrobial Resistance Patterns in Diabetic Foot Infections, an Epidemiological Study in Northeastern Italy

**DOI:** 10.3390/antibiotics10101241

**Published:** 2021-10-13

**Authors:** Giovanni Boschetti, Dino Sgarabotto, Marco Meloni, Marino Bruseghin, Christine Whisstock, Mariagrazia Marin, Sasa Ninkovic, Michela Pinfi, Enrico Brocco

**Affiliations:** 1 U.O. Piede Diabetico, Policlinico Abano Terme, 35031 Padova, Italy; mbruseghin@casacura.it (M.B.); cwhisstock@casacura.it (C.W.); mmarin@casacura.it (M.M.); sninkovic@casacura.it (S.N.); mpinfi@casacura.it (M.P.); ebrocco@casacura.it (E.B.); 2 Policlinico Abano Terme, 35031 Padova, Italy; dsgarabotto@casacura.it; 3 U.O. Medicina del Piede Diabetico, Università di Tor Vergata, 00133 Rome, Italy; meloni.marco@libero.it

**Keywords:** MRSA, diabetic foot infections, AMR, OPAT, EARS network

## Abstract

This study is a retrospective epidemiological assessment of bacterial species isolated from a cohort of out-patients with diabetic foot infections referred to our “Diabetic Foot Unit” over one year, with particular attention to index pathogens, as identified by the EARS Network. *Staphylococcus aureus* and *Pseudomonas aeruginosa* accounted for 33.5% and 11.9% of cases, respectively. *MRSA* was isolated in 27.1% of patients, with 14.06% showing additional resistance to three antimicrobial classes. *Pseudomonas aeruginosa* presented extensive resistance to fluoroquinolones (57.3%), which was associated with resistance to piperacillin in 17.6% or to carbapenems in 23.5% of cases. Other pathogens, such as *methicillin resistant* *Staphylococcus epidermidis*, *Escherichia coli* and *Morganella morganii* ESBL and *Enterococcus faecium* VRE, were also found.

## 1. Aim

The aim of this study was to gather baseline data about the antimicrobial resistance patterns of bacteria isolated from diabetic foot infection according to the WHO Anti-Microbial Resistance Global Report, EARS-Net guidelines and PNCAR 2017–2020 (Italian National Protocol for Contrast of Antimicrobial Resistance).

## 2. Background

Diabetes is a chronic disease afflicting more than 422 million people in the world [1]. In Italy, up to 5.3% of the total population suffer from diabetes, with an increased prevalence of 16.5% among people older than 65 years [2]. Diabetic foot ulcers are a major cause of morbidity, accounting for at least two-thirds of all non-traumatic amputations, with a survival rate of 5 years—far worse than the survival rate for both colon and breast cancer. The incidence of diabetic foot ulcers varies between countries, with a global average of 6.3% (5.1% in Europe [3]). Infected or ischaemic diabetic foot ulcers account for approximatively 25% of all hospital stays for patients with diabetes [4]. The estimated costs of diabetic foot ulcers differs vastly, depending on many variables, with studies calculating as much as double these costs for infections [5,6,7]. Of further importance, antimicrobial resistances have been steadily rising during the last 50 years and are among the critical “calls for action” by the WHO for the future [8]. Italy, according to the WHO Anti Microbial Resistance Global Report’s and the European Antimicrobial Resistance Surveillance Network’s (EARS-Net, ECDC) indications [9], has implemented a national protocol for the contrast of antimicrobial resistances (PNCAR [10,11]) but data regarding cutaneous wounds, even in international literature, are scarce [12,13,14]. This research is an epidemiological assessment of bacterial species isolated from a cohort of out-patients with clinically infected foot ulcers, as assessed according to IWGDF/IDSA guidelines [15,16] and PEDIS [17] classification, referred to our “Diabetic Foot Unit” between November 2018 and November 2019 from a catchment area of almost 1 million people in north-eastern Italy.

## 3. Material and Methods

A total of 441 microbiological tests of patients with a diagnosis of infected foot ulcers were collected between November 2018 and November 2019. Fifteen entries were excluded as non-diabetic patients with foot ulcers of different origin. The confirmatory diagnosis of infection was clinical for lesions of grade 2 or higher according to the PEDIS classification (equivalent to mild, moderate or severe infections, according to IDSA guidelines). A total of 426 consecutive microbiological reports from 340 diabetic patients (246 males mean age 69 years, 94 females mean age 73 years) were then considered for this study. Fifty-four patients had two different entries and sixteen had three. The vast majority of patients came from north-eastern Italy, within 100 km of our location. Clinically infected lesions underwent routine cleaning with 10% povidone water solution, followed by debridement, if deemed necessary. Punch biopsies of deep tissues (at direct contact with the bone in case of grade III as Texas University Classification [18]) were then collected and analysed by our hospital laboratory using the microdilution broth method Vitek 2 (BioMerieux, Florence, Italy [19]). Antimicrobial analysis tested different resistance patterns accordingly to the isolated strain. In our statistical analysis we specified if the resistance was extended to the whole class or limited to single active principles. After a microbiological sample was collected, all patients were prescribed empiric antimicrobial therapy, if deemed appropriate by the physician (amoxicillin/clavulanate 875/125 mg 1 tab TID for 5 days; if allergic, the patient was prescribed trimethoprim/sulfamethoxazole 800/160 mg 1 tab BID or ciprofloxacin 500 mg 1 tab BID; patients with moderate or severe kidney disfunction had their dosages modified accordingly). After the microbiological results were available (5–6 days), all therapies were confirmed or modified accordingly with the antibiogram of the pathogenic bacteria. The duration of the therapy widely varied based on clinical response, previous therapies, patient compliance and severity of the infection but usually lasted for an average of 12 days, with a few exceptions among patients with multiple microbiological analyses. Patients with severe non-responding infections and life- or limb-threatening infections underwent hospitalization, intravenous antimicrobial therapy and surgical procedures aimed to save their foot or life. Microbiological culture results were ordered for pathogen number (one, two or three at simultaneously), and specific strain. Study variables included age, gender, pattern of antibiotic resistance and index pathogen frequency. Descriptive statistics methods were applied, to determine the distribution of frequencies, using Microsoft Excel 2019.

## 4. Results

Bacterial growth was detected in 406 samples (20 samples, 4.7% of which proved negative). Two-hundred and fourty-one (56.5%) showed growth of a single strain of bacteria, two strains grew in 146 samples (34.3%) and three strains in 15 samples (3.5%), whereas fungal growth alone or along with bacterial growth was found in 7 (1.6%) samples. Roughly, half of the positive samples (192) were collected from acute ulcers (present from less than 8 weeks). The pathogen resistance pattern is shown in Table 1; in fact, the bacteria isolated are mainly resistant to one or more than two classes of antibiotics. Only 15.7% of the *Staphylococcus aureus* strains were sensitive to all antibiotic classes; much worse for *Enterococcus faecium*, *Klebsiella pneumoniae* and *Enterobacter cloacae* strains, which showed, in all cases, some level of resistance, while *the Escherichia coli* and *Pseudomonas aeruginosa* strains displayed an absence of resistance in only 18.75% and 33.8% of samples, respectively. Table 2 highlights the multiple antimicrobial resistance of four most common pathogens (*Escherichia coli, Klebsiella pneumoniae, Pseudomonas aeruginosa* and *Staphylococcus aureus*). For example, *Escherichia coli* strains were resistant to both co-trimoxazole and ESBL in three samples, and to fluoroquinolones and ESBL in five samples. *Klebsiella pneumoniae* strains were resistant to carbapenems in two cases, with double resistance to fluoroquinolones but not to co-trimoxazole. *Pseudomonas aeruginosa* strains had a different pattern of double resistance: resistance to carbapenems were always (16/16) associated with resistance to fluoroquinolones but less to piperacillin (16/5), to ceftazidime/cefepime (16/4), to aminoglicosydes (16/2) and colistin (16/2). Lastly, *Staphylococcus aureus* strains, resistant to oxacillin (MRSA), were often refractory, even against fluoroquinolones (52/45) and MLSb (macrolide, lincosamide, streptogranin B: 52/29), while much less so to aminoglicosydes (52/19) and tetracycline (52/9). Finally, Table 3 lists the index pathogens found in our study. *Staphylococcus aureus* and *Pseudomonas aeruginosa* accounted for roughly half of the cases (respectively 33.5% and 11.9%) and showed widespread antimicrobial resistance (detailed data on Appendix A); MRSA was isolated in 52 cases (27.1%), while the other 27 samples (14.06%) showed resistance to three different antimicrobial classes without methicillin resistance. All (21) *Morganella morganii* were resistant to at least three different classes of antibiotic, and only isolated *Enterococcus faecium* (VRE pattern) showed resistance to vancomycin, teicoplanin, gentamicin, kanamycin, ampicillin and imipenem (detailed data on Appendix A).

## 5. Discussion

The Italian National Plan for the Contrast of Antibiotic Resistance (PNCAR, 2017) is an organic memorandum that contextualize the 2015 WHO resolution produced during the 68th world health assembly and regarding the rise and spreading of antimicrobial resistances. Our paper is a retrospective epidemiological assessment of bacterial species isolated in a cohort out-patients with clinically infected foot ulcers referring to our “Diabetic Foot Unit” within one year, with particular focus on antimicrobial resistance (AMR) patterns. Our data emphasize the spread of pathogens resistant to multiple antibiotics making difficult the choice of an effective therapy. Diabetes is a well-known risk factor for the development of infections frequently characterized by atypical localization, severity and difficult pathogens. A close relationship between foot infection, amputation and mortality had been thoroughly studied [20,21]. Furthermore, age, high comorbidity and severity indexes of diabetic patients are all factors that increase the risk of antibiotic misuse and de facto limits the suitability of several active principles [22]. In this frame, high resistance rates to commonly used antimicrobials, especially for *Staphylococcus aureus* and *Pseudomonas aeruginosa*, imply further treatment difficulties and worst prognosis [23]. Our data confirm that it is not uncommon to encounter other index pathogens usually found only in the Intensive Care Units. The importance of AMR is particularly relevant when dealing with indications to antimicrobial treatment and the decision to treat as outpatients. The first topic is thoroughly analysed in the IWGDF guidelines [16] and different studies have ascertained the beneficial effects of implementing practical guidelines for microbiology, and on costs and outcomes [24,25]. With regard to outpatient treatment, severe or non-responding ulcers should be referred to second- and third-level diabetic foot units as outpatients with limb- or life-threatening wounds and immediately admitted. During this phase, antimicrobial therapy is almost mandatory and the general practitioner should prescribe empirically the drug and then clinically monitor the patient through out-patient setting or house-calls. When antimicrobial therapy is indicated and oral administration is not feasible due to resistance patterns, allergies or specific comorbidities, intra-venous home therapy may be considered under specific conditions (Outpatient Parenteral Antibiotic Therapy OPAT [26]). The antibiotic treatment of diabetic foot ulcers remains a key step in the therapeutic process, even if all these factors impose such a selection pressure on the skin microbiota that the rise of multi- and pan-resistant strains becomes, typically, but a matter of time.

### Study Limitations

(a) A lack of precise information regarding previous antimicrobial therapy;

(b) Being a third-level Diabetic Foot Unit, our study population was shifted towards more complicated lesions and infections.

## 6. Conclusions

Baseline data on AMR in diabetic foot infections are still scarce and fragmented both geographically and quantitatively; an epidemiological map of pathogens and resistance patterns would be of paramount importance both in terms of costs and outcomes. Our study points out the spread of multi-resistant pathogens in diabetic foot infections, making mandatory the acquisition of a bacterial culture before any effective antibiotic therapy. An empiric, single-agent, antimicrobial therapy prescribed for the “average” diabetic foot ulcer must face difficult pathogens that resist at least two different antibiotic classes in more than 50% of cases. Proper sampling, debridement and medications, timely microbiological analysis and adequate antimicrobial therapy are key steps in the therapeutic process and require collaboration among different healthcare professionals and health services.

## Figures and Tables

**Table 1:** Resistance pattern of our pathogens distinguishing the strains resistant to one class of antimicrobials from those resistant to 2 or more classes.

Bacteria	No Resistances		R to 1 Class		R ≥ 2 Classes		Total	
	n	%	n	%	n	%	n	%
*S. aureus*	30	15.7	64	33.3	98	51	192	33.5
*S. coagulase-*	6	10.2	7	11.9	46	77.9	59	10.3
*E. faecium*	0	0	0	0	1	100	1	0.17
*E. faecalis*	20	55.6	9	25	7	19.4	36	6.2
*Streptococci*	10	27	18	48.6	9	24.4	37	6.4
Other gram+	3	30	2	20	5	50	10	1.8
*E. coli*	6	18.75	1	3.125	25	78.125	32	5.6
*K. pneumoniae*	0	0	0	0	8	100	8	1.4
*Acinetobacter spp*	3	50	0	0	3	50	6	1.03
*P. aeruginosa*	23	33.8	16	23.6	29	42.6	68	11.9
*E. cloacae*	0	0	3	11.5	23	88.5	26	4.5
*M. morganii*	0	0	0	0	21	100	21	3.8
*S. marcescens*	0	0	0	0	19	100	19	3.3
*P. mirabilis*	0	0	1	6.7	14	93.3	15	2.6
*K. oxytoca*	1	11.1	5	55.6	3	33.3	9	1.6
Other gram–	8	23.5	5	14.7	21	61.8	34	5.9
Total	110	19.2	131	22.9	332	57.9	573	100

**Table 2:** Description of double resistance against antimicrobials for the four most common index pathogens; the number at the intersection of columns and rows indicates the double resistance to the specified antibiotics (absolute numbers and percentages). Oxacillin resistance predicts resistance also to cephalosporines, carbapenems, beta-lactams.

***S. aureus* 192**	**MRSA**	**Oxacillin**	**Fluoroquinolones**	**Aminoglycosides**	**MLSb**	**Tetracycline**
MRSA	52 (27.1%)	52 (27.1%)	45 (23.4%)	19 (9.9%)	29 (15.1%)	9 (4.7%)
oxacillin	52 (27.1%)	52 (27.1%)	45 (23.4%)	19 (9.9%)	29 (15.1%)	9 (4.7%)
fluoroquinolones	45 (23.4%)	45 (23.4%)	59 (30.7%)	17 (8.8%)	34 (17.7%)	5 (2.6%)
aminoglycosides	19 (9.9%)	19 (9.9%)	17 (8.8%)	25 (13%)	14 (7.3%)	11 (5.7%)
MLSb	29 (15.1%)	29 (15.1%)	34 (17.7%)	14 (7.3%)	51 (26.6%)	9 (4.7%)
Tetracycline	9 (4.7%)	9 (4.7%)	5 (2.6%)	11 (5.7%)	9 (4.7%)	21 (10.9%)
***P. aeruginosa* 68**	**Piperacillin**	**Ceftazidime Cefepime**	**Carbapenems**	**Fluoroquinolones**	**Aminoglycosides**	**Colistin**
penicillins	14 (20.6%)	10 (14.7%)	5 (7.3%)	12 (17.6%)	5 (7.3%)	3 (4.4%)
ceftazidime cefepime	10 (14.7%)	12 (17.6%)	4 (5.9%)	10 (14.7%)	4 (5.9%)	3 (4.4%)
carbapenems	5 (7.3%)	4 (5.9%)	16 (23.5%)	16 (23.5%)	2 (2.9%)	2 (2.9%)
fluoroquinolones	12 (17.6%)	10 (14.7%)	16 (23.5%)	39 (57.3%)	6 (8.8%)	5 (7.3%)
aminoglycosides	5 (7.3%)	4 (5.9%)	2 (2.9%)	6 (8.8%)	7 (10.3%)	3 (4.4%)
colistin	3 (4.4%)	3 (4.4%)	2 (2.9%)	5 (7.3%)	3 (4.4%)	8 (11.8%)
***E. Coli* 32**	**Penicillins**	**Cephalosporines**	**Carbapenems**	**Fluoroquinolones**	**Co-trimoxazole**	**ESBL**
penicillins	24 (75%)	6 (18.7%)	1 (3.1%)	12 (37.5%)	10 (31.2%)	5 (15.6%)
cephalosporines	6 (18.7%)	6 (18.7%)	0	5 (15.6%)	3 (9.4%)	5 (15.6%)
carbapenems	1 (3.1%)	0	1 (3.1%)	0	0	0
fluoroquinolones	12 (37.5%)	6 (18.7%)	0	15 (46.9%)	7 (21.9%)	5 (15.6%)
co-trimoxazole	10 (31.2%)	4 (12.5%)	0	7 (21.9%)	12 (37.5%)	3 (9.4%)
ESBL	5 (15.6%)	5 (15.6%)	0	5 (15.6%)	3 (9.4%)	5 (15.6%)
***K. pneumoniae* 8**	**Penicillins**	**Cephalosporines**	**Carbapenems**	**Fluoroquinolones**	**Co-trimoxazole**	**ESBL**
penicillins	7 (87.5%)	4 (50%)	2 (25%)	5 (62.5%)	4 (50%)	3 (37.5%)
cephalosporines	4 (50%)	4 (50%)	2 (25%)	3 (37.5%)	2 (25%)	3 (37.5%)
carbapenems	2 (25%)	2 (25%)	2 (25%)	1 (12.5%)	0	1 (12.5%)
fluoroquinolones	5 (62.5%)	3 (37.5%)	1 (12.5%)	5 (62.5%)	4 (50%)	3 (37.5%)
co-trimoxazole	4 (50%)	2 (25%)	0	4 (50%)	4 (50%)	2 (25%)
ESBL	3 (37.5%)	3 (37.5%)	1 (12.5%)	3 (37.5%)	2 (25%)	3 (37.5%)

**Table 3:** Index pathogens—as indicated in PNCAR 2017—found in the present study.

Index Pathogens	ESBL	VRE	MLSb	MRSA	Carbapenemase Producer	MDR (≥2 Classes)	Total
*Escherichia coli*	5 (87.5%)					25 (78.1%)	32
*Pseudomonas aeruginosa* MDR						29 (42.6%)	68
*Klebsiella pneumoniae*	3 (37.5%)				2 (25%)	8 (100%)	8
*Acinetobacter* spp. XDR					3 (50%)	3 (50%)	6
*Staphylococcus aureus*			28 (14.6%)	52 (27.1%)		98 (51%)	192
*Enterococcus faecium*		1 (100%)				1 (100%)	1

## Data Availability

The data presented in this study are available on request from the corresponding author. The data are not publicly available due to privacy reasons.

## Appendix A

Descriptive report of antimicrobial resistances.

Legend: R = resistant; penicillins = ampicillin, piperacillin, piperacillin + Tazobactam; cephalosporines = ceftazidime, cefepime, cefazoline; fluoroquinolones = ciprofloxacin, levofloxacin; carbapenems = imipenem, meropenem, ertapenem; aminoglycosides = gentamicin, amikacin, Tobramycin. ESBL = extended spectrum β-lactamase producer; KPC = *Klebsiella* carbapenemase producing; MLSb = macrolide, lincosamide, streptogranin B resistant; MRSA = methicillin resistant *Staphylococcus aureus.*

***Enterococcus faecium***: -1 isolated R to everything except linezolids (gentamicin, vancomycin, teicoplanin, kanamycin, ampicillin, imipenem)
***Enterococcus faecalis*** 36 isolated: -10 R high dose gentamicin and kanamycin; 5 of which also R to trimethroprim/sulfametoxazole -4 R to trimethroprim/sulfametoxazole only -2 R to trimethroprim/sulfametoxazole and cephalosporin (ceftaroline)
***Staphylococci* coagulase negative** 37 isolated: -29 R to at least 1 antibiotic class -26 R to multiple antibiotic classes (3 classes or more in 23 cases) and to methicillin -10 MLSb of which 9 also R to methicillin
***Streptococci*** 37 isolated: -*S. agalactiae* 25: 2 MLSb; 2 R to high dose kanamycin and gentamicin; 17 R to tetraciclin -*S. anginosus* 4: no resistances -*S. constellatus* 2: no resistances-*S. dysgalactiae* 5: 3 MLSb; 2 R to Tetraciclin -*S. mitis* 1: R to levofloxacin
***Staphylococcus aureus*** 192 isolated: -30 showed no resistances -53 R to penicillins only -11 R to only 1 antibiotic class other than penicillins (5 R to aminoglycosides, 2 R to macrolides, 1 R to trimethroprim/sulfametoxazole, 1 R to fluoroquinolones, 1 R to Mupirocine, 1 R to Fusidic acid)-19 R to 2 classes (13 R to penicillins and one among aminoglycosides, fluoroquinolones, trimethroprim/sulfametoxazole, tetracicline, fusidic acid; 5 R to macrolides and licosamides; 1 R to tetracicline and aminoglycosides) -27 R to at least 3 different classes but not showing methicillin resistance (22 R to penicillins + at least 2 other classes; 5 R to 3 different classes other than penicillins) -28 MLSb or MLSb inducible -52 R to multiple (>3) classes and methicillin resistant-MRSA (8 also R to trimethroprim/sulfametoxazole; 1 also R to Linezolid)
***Staphylococcus epidermidis*** 22 isolated: -22 R to at least 1 antibiotic class -11 MLSb -17 methicillin R -10 MLSb and methicillin R
**Other gram**+: 10 (*C striatum* 3; *S lugdunensis* 3; *S intermedius* 2, *F magna* 1, *S capitis* 1)
***Escherichia Coli*** 32 isolated: -25 resistant to at least 2 antibiotic classes -5 ESBL of which 3 also R to fluoroquinolones and trimethroprim/sulfametoxazole; 2 also R to fluoroquinolones -24 R penicillins (ampicillin 24 R; piperacillin 21 R) of which 1 also R to ertapenem; 1 also R to fluoroquinolones and trimethroprim/sulfametoxazole; 5 also R to fluoroquinolones; 5 also R to trimethroprim/sulfametoxazole -15 R fluoroquinolones 2 of which also R to trimethroprim/sulfametoxazole
***Klebsiella pneumoniae*** 8 isolated: -6 showed resistance to at least 3 different classes of antibiotics (4 R to penicillins, fluoroquinolones and trimethroprim/sulfametoxazole; 2 KPC) -3 ESBL (all R also to fluoroquinolones and 1 R to fluoroquinolones and trimethroprim/sulfametoxazole) -7 R penicillins -5 R fluoroquinolones
***Acinetobacter*** spp. 6 isolated: -2 *A. baumanii* (1 MDR carbapenemase producing R to everything except colistin) -4 *A. iwoffii* (2 resistant to 3rd gen cephalosporines and ertapenem)
***Enterobacter cloacae*** 26 isolated: -26 R to amoxicillin/clavulanate and ampicillin -5 R to > 3 antibiotic classes and carbapenemase producers -1 ESBL
***Pseudomonas aeruginosa*** 68 isolated: -45 R to at least 1 antibiotic class -12 R to fluoroquinolones only -10 R to fluoroquinolones and carbapenems only-2 R to fluoroquinolones and penicillins only-2 R to fluoroquinolones and cephalosporines only -2 R to penicillins and cephalosporines only -1 R to fluoroquinolones and aminoglycosides only -1 R to aminoglycosides only -1 R to fluoroquinolones and colistin only -3 R to colistin only -1 panresistant (R to everything except imipenem mic 1 mg/L)
***Morganella morganii*** 21 isolated: -21 R to at least 3 antibiotic classes -20 R to ampicillin and amoxicillin/clavulanic acid -11 R to fluoroquinolones -10 R to sulfatrimetrophim
***Serratia marcescens*** 19 isolated: -17 R to amoxicillin/clavulanic -12 R to at least 3 antibiotic classes
***Proteus mirabilis*** 15 isolated: -14 R to at least 3 antibiotic classes -9 R to fluoroquinolones and trimethroprim/sulfametoxazole
***Klebsiella oxytoca*** 9 isolated: -8 R to ampicillin -3 R to at least 3 antibiotic classes
**Other gram**–: 34 (*C. freundii* 2; *C. koseri* 2; *C. braaki* 1; *E. aerogenes* 2; *P. putida* 4; *P. rettgeri* 6; *P. vulgaris* 5; *S. maltophilia* 5; *A. xylosoxydans* 2; *A. faecalis* 2; *Enterobacter* spp. 1; *P. bivia* 1; *P. stuartii* 1)
**Fungi**: 7 (*Aspergillus* spp. 1, *C. albicans* 1, *C. parapsilosis* 2, *T. metagrophytes* 2, *T. rubrum* 1)
